# Epistemicide and colonial knowledge: a contrapuntal reading of Nair historiography in South India

**DOI:** 10.3389/fsoc.2026.1907593

**Published:** 2026-09-04

**Authors:** S. Anandhu, C. V. Anupama Nayar, A. S. Aneesh

**Affiliations:** 1 Department of English and Cultural Studies, CHRIST (Deemed to be University), Bangalore, India; 2 Department of Commerce, Kerala University, Trivandrum, India

**Keywords:** contrapuntal reading, decolonial historiography, emplotment, epistemicide, Kerala, matriliny, Nair community

## Abstract

Colonial power in South Asia operated not only through territorial administration but also through the systematic codification of indigenous societies into Western epistemic categories. This article investigates the historiographical construction of the Nair community in Kerala, South India, specifically focusing on the matrilineal institutions of *marumakkathayam* and *sambandham*. Situating the study within debates on colonial epistemology and postcolonial theory, the article analyses how ethnographic and historical narratives emplot Nair social institutions within Eurocentric interpretive frameworks. Drawing on Boaventura de Sousa Santos’s concept of epistemicide, Bernard Cohn’s theorisation of colonial forms of knowledge and Edward Said’s method of contrapuntal reading, the article traces how imperial discourse framed indigenous social practices into reductive binaries of “oppressor” and “oppressed”. The study advances debates in decolonial historiography by exposing the narrative structures through which colonial knowledge systems produced and perpetuated epistemicide. This contributes to the ongoing efforts to decolonize the study of South Asian communities.

## Introduction

Historical production under colonial rule was inseparable from the power structures that determined how colonised societies were perceived and categorised. Bernard (Cohn (1996, pp. 3–11) account of the British “investigative modalities” in India (the historiographic, the enumerative, the survey, and the museum) identifies the institutional procedures through which colonial administrations translated indigenous social practice into categories legible to European intellectual frameworks. Such historiographical bias was not incidental to individual historians but structurally produced: the language and semantic tools used to record history introduced skewed framings shaped by dominant power structures and by colonial or nationalist ideology (Bhat et al., 2023, pp. 698-705). As (Chakrabarty, 2008, pp. 27-46) argues, Western historians imposed their own categories onto non-Western histories, subordinating local pasts to a universalised European trajectory.

This translation operated through an Orientalist logic in which Western scholarship constructed the “orient” as irrational and unchanging against a “civilized” West (Said, 1978, p. 40), and through which colonial rule hardened a fluid system of social bonds into the modern, fixed institution of caste (Dirks, 2001). Müller’s (1859, p. 31) *A History of Ancient Sanskrit Literature* exemplifies this dichotomy especially framing “Greece and India” as “the two opposite poles” of human historical development, Müller cast Greek existence as materially engaged and Hindu existence as mere “illusion,.” This essentializes the East as fundamentally passive against a West defined by its command of material reality. Reproduced in missionary and ethnographic literature, this Orientalist grammar subjected indigenous kinship, marriage, and inheritance to the scrutiny of Victorian morality and social Darwinism, reframing them as “deviant” customs requiring imperial reform. These epistemic structures persist as a psychological and intellectual reality in postcolonial India, sustained by taxonomies that were, at times, adopted and disseminated by Indic researchers themselves. Santos (2014, p. 92) terms this process “epistemicide.” Epistemicide is the systematic destruction or delegitimisation of ways of knowing outside the European tradition. Its effects are visible across the historiography of numerous Indian communities, colonial and postcolonial alike.

A primary subject of this ethnographic gaze was the Nair (or Nayar) community of Kerala, whose matrilineal institution of *marumakkathayam* and consensual marital custom of *sambandham* presented a social logic that European observers found both intriguing and difficult to categorize. The Nairs were, and remain a large caste cluster of Kerala. Nairs were historically a martial and landholding class, providing soldiers, palace officials, and militia to Kerala’s ruling houses, and standing as *jenmis* (landholders) within the region’s agrarian order (Fuller, 1976). Ritually subordinate to the Nambudiri Brahmins, Nairs were nonetheless dominant over the great majority of Kerala’s population in economic and political terms prior to colonial rule, and internally stratified into a large number of sub-groups differentiated by occupation, territorial attachment, and ritual rank, a stratification documented in unusual granular detail in the colonial census record itself, which lists dozens of named Nair sub-castes under a single administrative head (Nagam Aiya, 1884). It is this combination, a regionally dominant, internally differentiated caste whose social organization nonetheless departed sharply from the patrilineal, patriarchal norm assumed by both the pan-Indian *varna* schema and by European kinship categories, that made the Nairs simultaneously so consequential a subject for colonial governance and so persistent a site of colonial and postcolonial misrecognition.

Matriliny was not unique to the Nairs: it was practiced, in varying forms, by the Tiyas and Mappilas of Kerala, the Bunts of coastal Karnataka, and the Khasi and Garo of Meghalaya—a regionally distributed kinship form that colonial scholarship persistently misread as anomalous rather than as one instance of a broader matrilineal complex (Schneider and Gough, 1961, pp. 3–29). The Nairs nonetheless received the most sustained colonial ethnographic attention, a disproportionate focus shaped by their dominant position within Kerala’s agrarian and military hierarchies, the visibility of *sambandham* to European observers, and the incompatibility of *marumakkathayam* with the patrilineal, property- consolidating logic of British administration. Within the *taravad*, the matrilineal ancestral household, formal ownership was vested in the female lineage while the karnavar exercised managerial authority, it is a configuration of female inheritance and male administration that colonial ethnographers, and the marriage-reform legislation that followed from their reports, repeatedly misread as social dysfunction rather than a coherent and stable order (Saradamoni, 1999, pp. 12–34; Kodoth, 2001, pp. 349–384; Arunima, 2003, p. 28). It is this specific misreading, and its transmission into postcolonial and indigenous historiography, that the present study investigates.

It is worth being explicit about why *marumakkathayam* and *sambandham*, rather than the full range of Nair social and political life, are treated here as the diagnostic site of epistemicide. Nair identity in the pre-colonial and colonial periods encompassed a great deal beyond kinship: a martial tradition organized around the kalari and the *charnavar* militias of the *taravad*-holding aristocracy, service as soldiers and palace officials to Kerala’s ruling houses, standing as *jenmis*(landholders) within the agrarian order, and a ritual position as temple functionaries subordinate in varna terms to the Nambudiri Brahmins but dominant in practice over the majority of Kerala’s population (Fuller, 1976). None of this falls outside Nair history, and a fuller social history of the community would need to engage all of it. The present study isolates *marumakkathayam* and *sambandham* because they were, specifically, the institutions on which the colonial ethnographic and administrative gaze concentrated its moralizing attention, that is the site where as the Introduction has argued, Victorian categories of kinship, legitimacy, and female propriety collided most directly with an indigenous social logic that had no equivalent in the European frameworks colonial observers brought to it. Marumakkathayam and *sambandham* are therefore not treated here as coextensive with Nair historiography as a whole, but as the specific institutional pressure point at which the mechanism of epistemicide this paper traces is most legible and best documented in the sources under analysis.

Existing scholarship on the Nairs has concentrated overwhelmingly on the structural mechanics of kinship and on the socio-political pressures behind the decline of *marumakkathayam* in the late nineteenth and early twentieth centuries (Kodoth, 2001; Jeffrey, 1976). Comparatively little attention has been paid to how the community itself was represented within colonial, postcolonial, and neocolonial historiography. It is an omission that has allowed early colonial misrepresentation to persist unexamined. Addressing this gap, the present study asks: how were the Nairs misrepresented by colonial historiographers, and through what mechanisms has this distortion been transmitted into, and internalized by, later indigenous historical writing? Answering this requires situating the Nair case within Quijano’s (2000, pp. 533–580) concept of the “coloniality of power,” the durable hierarchy of knowledge, outlasting formal empire, in which Western categories retain authority as the universal standard against which indigenous social forms continue to be measured.

To trace this transmission, the study undertakes a genealogical rather than comprehensive analysis of three texts, selected as representative nodes in a chain of epistemic transmission rather than as an exhaustive survey of Nair historiography, a survey that would necessarily engage Arunima’s (2003) *There Comes Papa*, Fuller’s (1976) *Nayars Today*, and the feminist historiography of Devika and Velayudhan. Fawcett’s (1901) *The Nayars of Malabar* is read as the originating colonial taxonomy, Jeffrey’s (1976) *The Decline of Nair Dominance* as its institutionalization within Euro-American South Asian studies, despite appearing in the post-independence period; and Peter and Peter’s (2006) *The Liberation of the Oppressed* as a case of its internalization within indigenous scholarship. Following Foucault’s conception of the archive as a site of power-knowledge, the question guiding this selection is not which texts are most authoritative but which chain of influence most clearly illuminates the mechanism of epistemicide; Arunima’s (2003) archivally grounded account of matriliny’s colonial transformation remains the scholarly standard against which the present argument is calibrated throughout. To analyze this transmission, the study employs Edward Said’s method of “contrapuntal reading”:

“We must therefore read the great canonical texts with an effort to draw out, extend, give emphasis and voice to what is silent or marginally present or ideologically represented in such works. The contrapuntal reading must take account of both processes, that of imperialism and that of resistance to it, which can be done by extending our reading of the texts to include what was once forcibly excluded” (Said, 1993, p. 74).

A contrapuntal reading brings to light the marginalized and silenced voices of the native community submerged within texts authored by Western historiographers, exposing the power dynamics between colonizer and colonized. As Menon (2015, pp. 64–83) observes, colonial history-writing treated Indian oral tradition as fable and legend, imposing a European standard of “truth” that left no room for lived indigenous experience. It is a structure of address that the following sections trace across Fawcett’s ethnography, Jeffrey’s monograph, and Peter’s indigenous revisionism.

### Nair community in colonial discourse

Fawcett’s *The Nayars of Malabar* was one of the first major efforts by a colonial official to record the traditions and social life of the Nairs in Kerala. Writing at a time when colonial anthropology was shaping how the world was understood, Fawcett looked at traditions like *marumakkathayam* (matriliny) and *sambandham* through the lens of European values. This approach often turned indigenous customs into “problems” that needed to be explained using Western ideas of family and morality. Such interpretations reflect what Said conceptualizes as Orientalist discourse, in which Western scholarship constructs the East as irrational, morally lax, and socially backward in contrast to the supposedly rational and progressive West (Said, 1978, p. 41). The colonial misunderstanding of *sambandham* also emerged from a fundamental unfamiliarity with matrilineal inheritance systems, in which property and lineage were traced through the female line rather than the paternal one. The female autonomy inherent in *marumakkathayam* presented a radical subversion of the Victorian gender hierarchy, where domesticity was strictly regulated by male authority. Moothedath argues that this agency was a source of profound colonial anxiety (Moothedath, 2015, pp. 32–36). Fawcett’s resulting ethnography serves as a case study in the “moral mapping” of empire, where indigenous kinship was reframed as a deviant social form, effectively exoticising the Nair woman while delegitimizing her social standing (Fawcett, 1901, p. 30).

The epistemic distortions identified in Fawcett’s ethnography are not confined to his interpretive framework alone but are traceable to the administrative conditions of his fieldwork. Fawcett served as Superintendent of Police in Malabar during the period of his ethnographic investigations, a role that structured his access to Nair informants and shaped the questions he brought to the data. Colonial administrative records held at the Indian Office Records (British Library, IOR/R/2) document the institutional context in which such ethnographic surveys were commissioned. The district collectors and police superintendents were periodically tasked with producing reports on “local customs” as part of the imperial intelligence apparatus (India Office Records, n.d.). The ethnographic survey was in Cohn’s terms, simultaneously an investigative modality and a governance technology, which is a procedure for producing knowledge that would be useful to the administration of colonial territories. Fawcett’s positioning as both observer and administrator meant that the “customs” he documented were always already filtered through the administrative requirement to classify, manage and ultimately reform the communities under his jurisdiction. This institutional embeddedness is what Santos would identify as the structural precondition of epistemicide. The destruction of indigenous knowledge does not require malicious intent but is the systemic outcome of an apparatus designed to translate social reality into administrative legibility.

Fawcett’s ethnographic discourse exemplifies the colonial project of moral policing, where the *sambandham* system was stripped of its cultural logic and recategorized as “concubinage”. This semantic reduction served to frame the Nair community as a deviant social subject. Fawcett explicitly links these kinship patterns to an evolutionary deficiency, arguing that polyandry represents a “survival of primitive social conditions” that remains fundamentally “inconsistent” with Western benchmarks of “advanced civilization” (Fawcett, 1901, p. 40). Fawcett’s ethnographic gaze moves from kinship to the sacred, characterizing Nair ritual life and moral disposition through a lens of colonial condescension. By labeling indigenous ceremonies as “wasteful” or “grotesque,” he engages in the classic imperial project of delegitimizing non-Christian spiritualities. This rhetoric serves to reinforce a binary where Western rationality is positioned against Eastern superstition, effectively “othering” the community’s entire metaphysical framework (Fawcett, 1901, p. 30). Labeling the Nair community as “primitive” and “uncivilized” represents a clear imposition of Western moral categories onto Eastern societies. This perspective aligns with the broader narrative of the “licentious East”, which contrasts a perceived moral laxity in the Orient with the Victorian values introduced by British colonists. Such a framework serves to legitimize colonial rule by characterizing the colonized as “primitive,” thereby justifying the necessity of a British “civilizing mission”.

An article by Mizutani discusses how colonialists applied the trope of the “licentious East” even to their own conduct. He argues that their characteristically “uncivilized” behavior (manifested in drinking, “loafing”, prostituting and so on) were allegedly exacerbated by the physical and cultural environment of India. According to Mizutani, this environment was believed to cause the mental and corporeal constitutions of Europeans to “degenerate”, necessitating strict racial and moral boundaries to maintain imperial authority (Mizutani, 2011, p. 206). Fawcett’s ethnographic narrative characterizes indigenous rituals through the dual tropes of irrationality and wastefulness. This dismissive stance toward non-Christian traditions functions as an “othering” mechanism, reducing complex Eastern spiritualities to “heathen” superstitions. This linguistic framing of rituals as “useless” established a persistent historiographical bias, influencing later scholars to replicate these colonial taxonomies. Ultimately, Fawcett’s work represents a calculated epistemic dismissal rather than a simple lack of information, serving to reinforce the moral superiority of the colonial center.

The distortions identified in the Nair context are symptomatic of a pervasive Western approach to the Eastern past. This study utilizes Hayden White’s “Metahistory” to deconstruct the underlying structures of Fawcett’s narrative. Through the process of “emplotment”, Fawcett organizes disparate ethnographic facts into a coherent story that reinforces colonial tropes. This theoretical lens reveals that the “history” produced is as much a product of linguistic and poetic choices as it is of empirical observation. For decades, Fawcett’s anthropology maintained the prestige of a rigorous scientific study before its inherent misrepresentations were critically challenged. Hayden White dismantled the assumption that history functions as a repository of absolute, undeniable facts, asserting instead that the discipline is always mediated by narrative structure. As Vann (1976) argues, historical discourse operates as a dual sign system: it points simultaneously toward the events it describes and toward a “generic story form” that provides the narrative with its formal coherence (Vann, 1976, pp. 151–167). In White’s view, historical accounts are not passively recorded; they are actively structured into narratives through the process of emplotment. These histories follow identifiable plot structures, where historians like Fawcett or Jeffrey adopt one of four distinct modes: tragedy, comedy, satire, or romance. Interestingly, White argues that this narrative structure, or emplotment, represents the most “manifest” or accessible level of a historical text, serving as the framework that gives historical data its specific generic shape (White, 1973, pp. 5–7).

Both Fawcett and Jeffrey portrayed the history of the Nair community through a tragic arc of “decline”. It is a narrative strategy obscured by the guise of a scientific anthropological study. Fawcett’s work reframed indigenous customs, specifically *sambandham* and matriliny, as barbaric relics persisting incongruously within a modernizing India. Utilizing a rhetorical register, he employed the community’s historical trajectory as a fundamental failure to achieve the benchmarks of Western civilization. Historiography begins with what White calls the pre-generic plot, the historian’s initial selection and ordering of facts that sets the stage for emplotment. The second element, emplotment, provides the structural framework by turning these facts into a recognizable story (White, 1973, pp. 5–7). Thus, Fawcett transformed the Nairs, their women, and their customs into characters tailored to his narrative arc. He characterized the Nairs as brave warriors whose social lives were marked by decadence. Thereby framing the community as a flawed central figure in the drama of civilizational decline. He depicted Nair women as symbolic figures embodying moral and sexual corruption, framing their traditions as grotesque spectacles that supposedly anchored society in a primitive state. This selective “emplotment” of history serves to legitimize imperial intervention. White’s (1973) tropology argues that historians rely on storytelling elements to structure their accounts with a specific plot, such as “rhetorical tropes”.

As a part of his historical emplotment, he deployed a range of metaphors to label polyandry’s “barbarism” and rituals as “waste”, while employing synecdoche to generalize the specific practices of a few individuals to the entire Nair community. He characterized the Nairs as exotic subjects of both fascination and scorn. Panikkar (1994) identifies Kerala as a site of exceptional historiographical complexity, arguing that the region’s early and unusually thoroughgoing agrarian reform movements, combined with a caste hierarchy in which ritual status and economic power were persistently misaligned (as in the Nair case itself). This produced a social field in which the gap between a community’s ritually-assigned position and its lived economic and political power was unusually visible to contemporaries and to historians alike. It is this specific misalignment, rather than institutional density in the abstract, that makes the epistemic effects of colonial representation both particularly visible and particularly consequential in the Kerala case (Panikkar, 1994, pp. 2597–2599).

The analysis offered here gains both confirmation and important qualification from Arunima’s landmark study of the colonial transformation of matriliny in Kerala. Drawing on legal records, colonial administrative correspondence and vernacular sources from Malabar and Travancore between 1850 and 1940, Arunima demonstrates that the reform and eventual abolition of *marumakkathayam* was not simply the outcome of missionary or British administrative pressure but was actively negotiated by Nair reform associations themselves. This is a process she characterizes as “the domestication of matriliny”. In her reading, the colonial archive does not straightforwardly impose a foreign patriarchal order. Rather, it intersects with the aspirations of a Nair middle class that saw the reform of *taravad* organization as a route to modernity and upward social mobility (Arunima, 2003, pp. 45–72). This complicates the present argument in a productive way, where the current study foregrounds the epistemicide wrought by colonial and neo-colonial scholarship. Arunima’s findings corroborates here that indigenous writers were not merely passive recipients of colonial discourse but active participants in its negotiation. The persistence of colonial tropes in the work of Peter and Peter must therefore be understood not as a simple mimicry of colonial authority but as what Homi Bhabha terms “colonial mimicry”. “Colonial Mimicry”, is a form of repetition that is almost the same but not quite, simultaneously affirming and subtly displacing the authority it reproduces (Bhabha, 1994, pp. 85–92). The distinction is analytically significant: epistemicide in this context, operates not only through external imposition but also through the internalization of colonial categories by those most directly defined by them.

The investigative modalities identified by Cohn (1996, pp. 3–11) which is the historiographic, the enumerative, the survey, and the museum, have so far been engaged at the level of theory. The enumerative modality can, however, be demonstrated directly. Nagam Aiya’s (1884) *Report on the Census of Travancore*, the printed record of the 1881 decennial census of the princely state of Travancore undertaken alongside the Imperial Census of India. It is a documentary instance of the apparatus Cohn describes, predating Fawcett’s ethnography by two decades and originating in a genre which is “routine colonial administration”, with no ethnographic pretension and no single author-observer whose individual bias might be held responsible for its distortions. Reading this document is instructive for what its silences and structural choices reveal about the mechanics by which an indigenous social order was rendered administratively legible. This is what Stoler (2009, pp. 1–20) calls reading “along the archival grain,” attending not to the facts a colonial document reports but to the categories, anxieties, and omissions built into its very form.

Nagam Aiya’s own introductory remarks on classification concede that the “377” minute sub-divisions of Hindu caste returned in the enumerator’s schedules were reduced, for tabulation purposes, to “81 larger groups” and then further to a final set of “45” heads submitted to the Imperial Census Commissioner, and that this collapsing exercise was undertaken with only the thinnest ethnographic warrant. As Nagam Aiya writes of the 377 sub-divisions: “In most cases the distinction is simply one of name, very little being known of the real differences between them in their manners or customs, laws of marriage or inheritance” (Nagam Aiya, 1884, p. 51). This is a remarkable admission to find in the government’s own record: the official compiler of the census states plainly that the caste categories being fixed into print were not derived from any working knowledge of the kinship or inheritance systems, precisely the domain, encompassing *marumakkathayam* and *sambandham*, that the present study is concerned with. The instructions reproduced later in the same chapter make the discretionary character of this process even clearer. Deputy Superintendents, the circular states, were “allowed a latitude in bringing together under the appropriate main caste such sub-divisions of caste as have been entered by the enumerators,” and were further permitted “to group together under one specific name any caste known in different parts of their province by different names” on the grounds that “local knowledge alone must be the guide” (Nagam Aiya, 1884, p. 178). What the census presents as a neutral count of pre-existing social facts was, on its own compiler’s testimony, an act of administrative consolidation performed by officials empowered to merge, rename, and standardize categories at their discretion. This is Cohn’s enumerative modality caught in the act: not recording a social order but manufacturing the terms in which it would thereafter be legible to the colonial state, and subsequently, to the historiography that took the census’s categories as its data.

The census’s occupational tables were compiled according to “Dr. Farr’s system” of occupational classification (Nagam Aiya, 1884, Table of Contents, Ch. VII). It is a taxonomy devised by the English statistician William Farr for the General Register Office of England and Wales, and built around the assumption of individuated, waged, typically male “occupations.” Applied to the Nairs, this taxonomy returns a striking asymmetry: of the community’s 143,317 recorded males, 82.54 per cent are returned as having an “occupation”; of 46,307 comparably enumerated females, only 19.71 per cent are (Nagam Aiya, 1884, p. 205). Where women’s occupations are recorded at all, they cluster overwhelmingly in service categories—36,811 as cooks and 1,599 as temple servants, against a comparatively small 1,740 returned under agriculture (Nagam Aiya, 1884, p. 206). While men’s recorded occupations span agriculture, government service, trade, medicine, law, and teaching. This is not merely a reflection of the sexual division of domestic labor; it is a structural artifact of the classification system itself. Under *marumakkathayam*, formal ownership of the *taravad’s* collective property was vested in the female lineage, even though day-to-day management was exercised by the karnavar (Saradamoni, 1999, pp. 12–34; Arunima, 2003, p. 28). This means that Nair women occupied, by birthright, the very position of proprietary authority that a Farr-derived occupational taxonomy, built for a wage-labor economy of individuated male breadwinners, has no category to register. A woman’s status as titular owner of an ancestral household and its lands does not appear anywhere in these tables, because the census’s conceptual grid was not designed to see it. The near-total absence of Nair women from the “occupation” columns is not evidence that they held no economic position; it is evidence that the colonial statistical apparatus could not represent the specific *kind* of economic position (proprietary rather than occupational, lineage-based rather than individually-waged) that *marumakkathayam* assigned to them. Read this way, the numbers themselves perform, quantitatively, the same erasure that Fawcett performs qualitatively when he reframes matrilineal female autonomy as moral deviance (Fawcett, 1901, p. 30): both render the actual structure of female authority within the *taravad* invisible, one through narrative condescension and the other through the blank cells of a statistical table.

The census’s appendix of the “377 min sub-divisions” of Hindu caste, given for “future guidance” and as “the basis of research from time to time as opportunities may offer” (Nagam Aiya, 1884, p. 178), lists thirty-six sub-divisions under the head “*Malayala Sudra*” alone (Nagam Aiya, 1884, pp. 179–186). Among these, set down as bare entries in a numbered list, with no gloss, no definition, and no indication of how they relate to one another, are terms that are not, in fact, caste names at all but functional and institutional titles internal to the *taravad* system: *Madambi*, denoting a Nair sub-group historically associated with estate management; *Panikkar*, a martial-instructional title; and thirteen separate compound entries carrying the suffix “Nair.” *Velatthu Nair, Puttathu Nair, Kadakathu Nair, Mala Nair, Mangalathu Nair, Kirinthu Nair, Pulikki Nair, Pada Nair, Nair*(unmodified), *Dikku Nair, Guru Nair, Vala Nair,* and *Malayathur Nair* (Nagam Aiya, 1884, pp. 182–184). Several of these designate not descent groups but roles or ranks that only make sense relationally, within the internal organization of the *taravad* and the wider structure of tenurial and martial obligation that bound Nair sub-groups to particular estates and temples. Set down as a flat, alphabetically-adjacent list of “castes” in a government ledger, this relational vocabulary is stripped of the institutional context that gave it meaning and reissued as a set of discrete, static, name-based identities. This is the same process, at the level of raw nomenclature, that Dirks (2001) describes at the level of the caste system as a whole: the hardening of “a fluid system of social bonds” into “a fixed and singular marker of identity.” Where Fawcett’s ethnography performs this hardening through narrative and moral judgment, the census performs the identical operation silently, through the sheer administrative act of listing.

Taken together, these archival findings do more than corroborate the paper’s Fawcett-centered account of colonial epistemicide; they extend it. They show that the systematic replacement of marumakkathayam’s institutional vocabulary and social logic with Western, Sanskritic, and statistical categories was not confined to the register of ethnographic narrative that Fawcett and, later, Jeffrey worked in. It was already embedded, two decades before Fawcett wrote, in the ostensibly neutral, numerical, bureaucratic register of the census. It is a genre that claims no interpretive authority at all and for that reason has never been read, by this paper’s own earlier draft included, as a site of epistemic violence. Reading the census’s tables and appendices against the grain of their apparent neutrality, in the sense subaltern historiography intends when it reads colonial administrative documents for what they inadvertently disclose rather than for what they set out to state (Guha, 1983). This makes visible an epistemicide that operated beneath the level of narrative altogether, in the categories, the discretionary latitude granted to enumerators, and the occupational taxonomy through which a matrilineal social order was made statistically legible to a colonial administration for the first time.

### Postcolonial representation of the Nair community in western scholarship

In *The Decline of Nayar Dominance*, Robin Jeffrey acknowledges the role of matriliny in shaping the historical trajectory of Kerala (Jeffrey, 1976, pp. 64-65). He observes that from 1500 to 1900, the European perspective on this system was characterized by a persistent tension between fascination and dismay, racing from the early accounts of Duarte Barbosa to the later anthropological work of Kathleen Gough. The gendered agency inherent in Kerala’s matrilineal social order, especially the freedom afforded to women in choosing their sexual partners in the past acted as an “orientalist magnet”, drawing Western scholars. Although Jeffrey’s work operates with a modern academic framework, it remains epistemically tethered to its colonial precursors. Uncritically adopting the terminology of 19th century officials, the text perpetuates a teleological view of history where modernization is synonymous with Westernization. His narrative often mirrors the missionary self-portrayal as benevolent liberators of lower castes, ultimately casting the British as the indispensable architects of social progress. Jeffrey incorporates a quote from the missionary Rev. Ebenezer Lewis to illustrate the perceived role of the church in colonial society.

“The poor, oppressed and trodden down natives…find that their grievances are more speedily, effectually and cheaply redressed by making the missionary their friend than in any other way” (Jeffrey, 1976, p. 59).

Utilizing such sources without a critical decolonial lens, his narratives reinforce the image of the missionary as a benevolent mediator and the primary source of justice for the colonized. Jeffrey’s reliance on missionary accounts (including the testimony of Rev. Ebenezer Lewis and similar London Missionary Society figures), without a robust counter-narrative drawn from indigenous sources or from the missionary archives themselves. This portrayal accepts the missionaries’ claim of altruistic sympathy for the low caste as an objective truth rather than a constructed persona. Jeffrey eventually admits that the missionaries were under strict orders from England to bring in as many converts as possible. Even after pointing out this clear motive, he quickly goes back to describing their work as an act of “genuine” kindness, making them look like noble heroes in the end (Jeffrey, 1976, p. 60). Thus, Jeffrey’s thematic focus on matriliny and Nair traditions further aligns his work with either the Fawcettian tradition.

Jeffrey’s analysis suffers from an epistemic limitation; by viewing the *taravad* through a Western prism. He dismisses its cultural coherence in favor of a narrative of obsolescence. He frames the indigenous kinship structure not as a resilient social institution, but as a liability in the face of global modernity. In his text, he mentioned it as the “Nair matrilineal joint-family” was destined for collapse, a failure Jeffrey attributes to the “concomitant” force of new economic resources and an uncompromising Western legal framework (Jeffrey, 1976, p. 17). The text characterizes the “rigorous legal system” imposed by the British as a neutral, modernizing force, largely decontextualized from its imperial origins. Making the *taravad* seem weak and outdated, Jeffrey makes its breaking apart seem like an unavoidable part of “progress”. Like other European writers before him, Jeffrey was drawn to Kerala because of its unique social structures, but he ended up relying on the same judgmental terms as Fawcett. Over time, these specific words and ideas became the standard way people talked about the Nair system, making it hard to see the *taravad* as anything other than a failure. The linguistic choices in *The Decline of Nair Dominance* reveal a deeply embedded colonial bias. In his narrative, the *taravad* is delegitimized through the use of pejorative terms such as “embarrassment” and “handicap” (Jeffrey, 1976, p. 129). This framing suggests that the matrilineal system was a roadblock to progress rather than a complex social organization. Presenting Western capitalism as the natural successor to the “encumbered” indigenous system, he validates a colonial hierarchy of values. This aligns with Hayden White’s argument that history is shaped by narrative devices and rhetorical tropes.

Jeffrey’s narrative of inevitable decline should be read through Chakrabarty’s (2008) critique of historicism. He criticized it as the assumption, embedded in European historical thought since the Enlightenment, that all societies pass through the same developmental stages at different speeds, with European modernity representing the universal endpoint. Chakrabarty identifies this as a “waiting room of history”. Non-western societies are perpetually positioned as temporally “behind”, occupying a historical stage that Europe has already passed through, and therefore requiring tutelage, reform, or intervention to advance (Chakrabarty, 2008, pp. 8–11). Jeffrey’s emplotment of Nair history instantiates this logic precisely. The *taravad* is not analyzed as a coherent social institution with its own rationality, it is framed as a historical survival. It is what Chakrabarty would call a “History 2”, a non-capital form of life that capitalism and modernity must eventually absorb or destroy. The “decline” of Nair dominance in Jeffrey’s account is thus not a neutral sociological observation but a deeply historicist narrative in which the Nairs are positioned as occupants of the waiting room. Their eventual displacement from social and economic power presented as the inevitable consequence of their failure to modernize along Western lines. This temporal othering, as Chakrabarty demonstrates, is not innocent, it is the epistemic mechanism through which colonial authority legitimized intervention in societies it framed as developmentally deficient.

Jeffrey’s work is less a neutral record and more a tragic emplotment that blames the community’s “decline” on its inability to shed its “outdated” cultural identity. The title’s use of the word “decline” shows that the book’s narrative is centered on the theme of loss (tragedy as emplotment). Even with a more academic approach, Jeffrey’s work relies on judgmental tropes like “decadence”, “inefficiency”, and “social embarrassment” (Jeffrey, 1976, pp. 17, 129). Fawcett’s colonial ethnography and Jeffrey’s neo-colonial historiography provide parallel but ideologically distant accounts of the Nair community’s transition. Though they overlap in subject matter and timeframe, their work is partitioned by their unique cultural environments (Jeffrey, 1976, p. 56). Nevertheless, Fawcett utilized a structure of moral superiority whereas Jeffrey focused on social modernization, with the result that both approaches placed the Nairs into a narrative that favored Western standards of civilization and progress. Despite their different contexts, both works represent a form of Western colonial intervention in Oriental history. In the view of sociologist Boaventur de Sousa Santos, colonialism was essentially “epistemicide”—the systematic destruction of rival knowledge through the domination of both a people and their intellectual traditions (Santos, 2018, p. 92). The concepts of Santos’s epistemicide and Said’s contrapuntal reading converge, illustrating the deliberate delegitimisation of indigenous knowledge system by colonial powers. The purpose of colonial historiography, as seen in the works of Fawcett and Jeffrey, is not merely to suppress voices but to fundamentally dismantle their epistemic foundations. As Santos argues, such “epistemicide” involves the “murder of knowledge”, where the domination of a people is completely the destruction of their capacity to define their own reality.

“Modern Western thinking is an abyssal thinking. It consists of a system of visible and invisible distinctions, the invisible ones being the foundation of the visible ones. The invisible distinctions are established through radical lines that divide social reality into two realms, the “this side of the line” and the “other side of the line”. The other side of the line vanishes as reality, becomes nonexistent, and is indeed produced as nonexistent. What most fundamentally characterizes abyssal thinking is thus the impossibility of the co-presence of the two sides of the line” (Santos, 2018, p. 118).

The systematic destruction of knowledge within this historiography manifests through four distinct stages: the delegitimization of indigenous knowledge of Nair systems, its replacement by colonial frameworks, the establishment of a new epistemic hegemony and its internalization by the native historians. Once local epistemologies are supplanted, colonial hegemony positions Western knowledge as the universal standard against which local communities are compelled to evaluate themselves. The colonized and post-colonial subjects begin to internalize and reproduce these external categories, reinforcing the very hegemony that marginalized their original identities. This internalization is epitomized by the community’s adoption of the derogatory phrase “fatherless marital system” to characterize *sambandham*. As Jeffrey notes that the *taravad* was increasingly viewed through a lens of “embarrassment”, a sentiment fueled by the specific taunt that “no Nayar knows his father” (Jeffrey, 1976, p. 129). This rhetorical move by missionaries successfully pathologised the matrilineal system by measuring against the patriarchal nuclear family. This taunt, emphasized by colonial and missionary authorities, eventually became a pervasive source of contempt for the entire Nair community. The hegemony of missionary discourse was legitimized and disseminated through both colonial and neo-colonial historiographies, a process that facilitated its internalization by the indigenous population, who gradually came to accept this revised history as their own.

Jeffrey’s periodization ends in 1908; his narrative of the *taravad’s* inevitable collapse is nonetheless offered as an explanation of a trajectory that continues well beyond that date. Tampi’s (1943) *Economic Survey, 1941*, the first sample-based economic survey conducted in Travancore, offers an independent, government-collected body of data from squarely within this later period, 16 years after the 1925 Travancore Nair Regulation legalized the partition of *taravad* property, against which the shape of Jeffrey’s “decline” can be tested rather than merely theorized against. The survey’s tables on family composition, broken down by community and income group, note a distinctive pattern among Nayar households: “the female adult preponderates in the first three income groups while in the income groups above Rs. 300 the number of female adults is less” (Tampi, 1943, p. 37). This is precisely the demographic signature one would expect of a matrilineal, natolocal household structure, women remaining within the natal *taravad* rather than relocating to a husband’s household on marriage, persisting 16 years after formal legal partition began. What makes this finding usable as evidence rather than mere suggestion is the survey’s own comparative method: in the same passage, the report *does* offer a sociological explanation for an analogous pattern among Brahman households, attributing the larger number of female adults there to women’s economic dependency and the “non-prevalence of remarriage” of widows, who remain “under the protection of the son or some other male relative” (Tampi, 1943, p. 37). No comparable explanation is offered for the Nayar case, the report simply records the pattern and moves on. This asymmetry is revealing in exactly the way this paper has argued colonial and neo-colonial documents tend to be revealing: the survey’s analytical vocabulary, built around a normatively patrilineal household in which women are dependants of male earners, has a ready explanation for female preponderance when it can be assimilated to that model (Brahman widowhood and dependency) and no explanation at all when the actual cause (matrilineal household structure) falls outside its conceptual repertoire. The *taravad*, in other words, is still statistically visible in 1941; what has been lost is not the pattern itself but the administrative vocabulary capable of naming it, 16 years into the very legal process that supposedly ended it.

The survey’s quartile tables of family indebtedness by community and purpose show the Nayar community carrying the highest, or near-highest, debt burden of any community surveyed across nearly every category: a median debt of Rs. 175 under “prior debts” against Rs. 101–160 for other communities, Rs. 159 under “investment” against Rs. 90–112 for others, and a median of Rs. 100 “for all purposes taken together” against Rs. 50–75 for Ilava, Christian, Muslim, and other communities (Tampi, 1943, p. 44). Read in isolation, and in the register Jeffrey and Peter write in, this would seem to confirm a narrative of Nair economic decline. Read against the specific legislative history of the preceding 16 years, it supports a materially different explanation. The 1925 Nair Regulation’s partition provision converted collectively-held, indivisible *taravad* property into individually partitioned shares (Arunima, 2003, pp. 45–72), a process that characteristically generates exactly the kind of debt this table records, inherited or “prior” debt from partition settlements and litigation, and new individual “investment” debt required to establish a viable smallholding where a joint household had previously sufflced. The Nayar community’s comparatively high debt burden in 1941 is therefore better read not as evidence that *marumakkathayam* was an institution in terminal, self-generated failure, as Jeffrey’s title and framing imply, but as the measurable economic residue of the specific legal instrument (colonially-pressured reform legislation) that dismantled it. The “decline” Jeffrey narrates as an internal inevitability appears, in this archival record, as a documentable legislative after-effect.

The survey’s ranking of communities against the state-wide standard of living complicates a uniform decline thesis further. “In the group below 60 (rupees per month), the Other Hindus, Nayar and the Muslim rank above the State level” (Tampi, 1943, p. 37); it is only in the higher income brackets that “the Nayar is able to reach the level of the State” only there, rather than exceeding it as the Brahman, Christian, and Muslim communities do (Tampi, 1943, p. 37). In other words, the poorest Nayar households in 1941 were doing comparatively better than the state average, while wealthier Nayar households lagged behind their Brahman, Christian, and Muslim counterparts. A single narrative of “decline,” pitched at the level of the community as a whole, cannot accommodate this internal stratification; it requires precisely the kind of disaggregated, class-differentiated account that Arunima’s (2003) archival work on the “domestication of matriliny” by an aspirational Nair middle class already gestures toward, and that the present data corroborates independently, from a different documentary source and 16 years later.

Taken together with the census evidence discussed above, these findings do not deny that the Nair community underwent significant, and in places genuinely difficult, economic and institutional change across the period this paper examines. What they contest is the specific narrative shape (a single, internally-generated, terminal decline) that Jeffrey’s emplotment imposes on that change, and that Peter’s later text inherits uncritically. The archival record instead shows a community whose matrilineal residential structure remained statistically legible 16 years after its legal dismantling began, whose recorded economic distress tracks closely with the timing and mechanics of that legal dismantling rather than with any internal institutional failure, and whose experience was materially stratified in ways a single declension narrative cannot represent.

### The internalization and reproduction of colonial epistemicide

Through the process of internalization, native historiographies both absorbed and subsequently reproduced this revised version of history. *The Liberation of the Oppressed: A Continuous Struggle* by Ivy Peter exemplifies this phenomenon with particular clarity. Framing matriliny as a fundamental aberration, Peter reproduces a narrative framework first established in Western historiography, directly aligning her work with Fawcett’s “primitive” stereotype and Jeffrey’s “embarrassment” thesis. The depiction of matriliny in such texts serves as a biased interpretation that mirrors colonial and missionary critiques, framing the system as a primary driver of social decay. Peter offers a definitive example of a native historian engaging in the internalization and revisionism of colonial narratives, particularly through her handling of the *sambandham* tradition, by explicitly reiterating the trope that “no Nair knows their father”, Peter directly reproduces an idea popularized in the neo-colonial scholarship of Robin Jeffrey.

“The most important pattern to be noted in the community of the Nairs was the system of inheritance. They followed the matrilineal system of inheritance…According to this system, my sister’s children were the inheritors. The Nair women were permitted to have marital relationship with any Nambudiri or Nair. Those Nambudiris or Nairs were not bound to care for the children born in such a relationship. Polyandry prevailed among them. As Robin Jeffrey observed, no Nair knew his or her father” (Peter and Peter, 2006, p. 25).

Beyond these specific tropes, Peter’s work functions as a site for the continued circulation of colonial teleologies and reproduces a broader constellation of notions inherited from colonial historiography. It perpetuates the imperial narrative of the “civilizing mission” by casting British officials and Protestant missionaries as the sole agents of progress and enlightenment. Within this framework, colonial intervention is portrayed as a necessary moral force that challenged “Nair dominance” and dismantled the community’s supposedly “barbaric” customs. According to Peter, the “selfless service” of protestant missionaries acted as the primary catalyst for social revolution, even overshadowing the substantial influence of British administrative rule (Peter and Peter, 2006, p. 35). The underlying subtext of the work serves as a modern revision of the “civilizing mission”, systematically characterizing pre-colonial society as inherently backward and barbaric, the narrative effectively validating the historical necessity of both colonial intervention and missionary governance. The linguistic shift from “influence” to “dominance” in the works of Jeffrey and Peter is central to their delegitimization of the Nair social system. The terminology labels the community’s historical position as an inherent injustice, making its “decline” seem like a moral imperative. Peter reinforces this by making the “Nadar revolt against the Nair domination” a central pillar of the narrative.

Recycling the vocabulary of the empire, Peter’s text functions as a contemporary vessel for the “civilizing mission” narrative. This illustration of crediting British governance and missionary ethics about the “enlightenment” is an extent of “entangled history”. The native historian no longer speaks from an independent epistemic position but from an internalized space where Western categories are used to measure the value of indigenous traditions. As defined by Bauer and Norton, entangled histories necessitate a rigorous examination of the “numerous sources, agencies, and direction of influence” that shape intercultural connectedness (Bauer and Norton, 2017, pp. 473–482). Despite their divergent temporal and cultural contexts, the colonial, neo-colonial, and post-colonial narratives of Fawcett, Jeffrey, and Peter are highly entangled. Each work reproduces and circulates the same derogatory stereotypes of the Nair community, demonstrating that “entangled history” is not a static relic of the past, but a continuous, evolving process of epistemic reinforcement. The “authentic way of knowing the Orient” has been rendered accessible because it became so deeply entangled with the colonial system that the two are now historically inseparable. This is a process that Peter and Peter’s text instantiates with particular clarity. Rather than a static outcome, Bauer and Norton (2017) define entanglement as any “unresolved and continuous process” of confrontations, shifts and revisions. The nature of historical entanglements are neither equal nor harmonious; they are fundamentally dictated by the asymmetrical power relations between colonial authorities and indigenous subjects. Ironically, the act of a native historian revising and documenting this entangled narrative is precisely what imbues it with a perceived authenticity for a broader audience. Through the dual forces of colonial intervention and the subsequent contributions of native authors, the history of the Nair community has become a complex site of misrepresentation and entanglement.

From the perspective of epistemicide, Fawcett is prominent among those who initiated the delegitimisation of Nair historiography. Colonial and neo-colonial scholars like Frederick Fawcett and Robin Jeffrey established hegemony, systematically replacing indigenous knowledge systems with Western sociological frameworks. This narrative was subsequently revised and internalized by native historians such as Ivy Peter, resulting in an entangled history for the community. While this entanglement is rooted in colonial discourse, achieving a clear understanding of the community’s past requires tracing these narratives back to their colonial origins, an intervention this research is performed for the Nair community of Kerala, south India. This binary social structure of “oppressor versus oppressed” established by Western colonial and Neo-colonial narratives finds a parallel in Hegel’s master–slave dialect. In *Phenomenology of Spirit* (1807), Hegel frames the relationship between the “Lord” and the “Bondsman” as a quest for self-consciousness that survives the initial “trail by death”. As Aching suggests, this Hegelian framework is essential for understanding how dependent self-consciousness is constructed under the gaze of a dominant power, a dynamic that underpins much of the historiography concerning Kerala’s social transitions (Aching, 2006, pp. 911–932). By reducing the Nair community to a “military caste” and pathologizing their kinship structures as “licentious”, Fawcett established the initial epistemic foundation for the “oppressor” archetypes. Jeffrey’s Neo-colonial intervention did not dismantle this trope. Rather, it reinvigorated it through the narrative of “declining dominance”, which fundamentally accepts the premise of an inherently oppressive past. This historiographical continuity has resulted in dialectical statements in Kerala’s historical writing and the entrenchment of their Western-constructed categories has ensured that the Nair identity remains locked in a binary dominance and decline.

Peter’s historiography adopts the Hegelian master–slave dialectics as its primary structural engine, framing Kerala’s social history as a teleological journey from feudal oppression to revolutionary enlightenment. The Nairs are essentialized as the stagnant “Lord”, while the Nadars occupy the role of the “Bondsman” who, through engagement with the material world and organized resistance, attains a superior self- consciousness (Aching, 2006, p. 915). This dialectic is not an organic indigenous development but a reproduction of the colonial “civilizing mission”, a root metaphor used by the British to delegitimize Nair authority by labeling it “enslavement”. By utilizing terms like “liberation” (of Nadar community from Nairs) in her title, Peter reinforces this manufactured dichotomy, demonstrating how colonial tropes have been internalized and rebranded as a revolutionary native discourse. The result is a historical narrative that remains locked within a Western-constructed binary of dominance and emancipation. This comparative framework reveals that the “authentic” history of the Nair community has been replaced by a series of overlapping Western-centric constructs. Fawcett established foundational stereotypes, Jeffrey refined these into an academic narrative and the process of epistemicide is completed in the work of Peter and Peter’s, where these external critiques are no longer viewed as foreign impositions but are adopted as native historical truths.

The analytical framework developed here finds further theoretical support in Anibal Quijano’s concept of the “coloniality of power”. This extends the argument from the specific case of Nair historiography to a structural account of how colonial hierarchies of knowledge persist in the postcolonial world. For Quijano, coloniality is distinct from colonialism as a historical period, colonialism ended with formal decolonisation and coloniality continues to structure social reality in the former colonies (Quijano, 2000, pp. 533–580). The persistence of colonial tropes in Jeffrey’s post-independence scholarship and their internalization in Peter’s indigenous revisionism are in Quijano’s terms, manifestation of coloniality rather than colonialism. They operate not through administrative command but through the continued authority of Western epistemological categories as the universal standard against which indigenous social forms are measured and found deficient. This conceptual extension is significant for the present argument because it shifts the analysis away from a critique of individual bias, which is the personal prejudice of a Fawcett or a Jeffrey, and toward a structural critique of the epistemic conditions that make such bias not only possible but systematically reproducible across different historical moments and different types of authors, including indigenous ones.

A question this argument must confront is whether the aversion to matriliny documented in Peter and Peter’s text is properly attributable to colonial epistemic inheritance at all. Alternatively, whether it reflects something more general: that any author formed by a modern, Western-derived education, a category that includes virtually every professionally trained historian writing in English in twentieth- and twenty-first-century India, colonized or not, will tend to reproduce categories of conjugal legitimacy, nuclear domesticity, and individuated property that did not originate in India regardless of the specific colonial history of any one community. Not every internalization of a Western normative category is *ipso facto* colonial in origin or mechanism; some may be better described as a general effect of global modernity’s institutional forms (like the school, the legal system, the university) that colonialism introduced but did not exhaust, and that continued to operate through independent postcolonial state-building long after 1947. The present study’s use of “native” and “indigenous” to describe Peter and Peter also requires more precise definition than it has so far received. Both authors write from within Kerala and from within a community, the Nadars, whose own history of caste subordination is central to their book’s argument; in this specific and limited sense (writing from inside the regional social field the book describes, rather than from the position of an external colonial administrator or metropolitan academic) they meet the working definition of “native” this study has used. This is a narrower claim than a claim to unmediated cultural authenticity, and the study does not intend the latter. It also raises the question of why Peter and Peter are the sole “native” text engaged here. The answer, consistent with the genealogical rather than comprehensive method set out above, is that they were selected as one legible instance of a broader phenomenon (the internalization of colonial historiographical categories within indigenous scholarship) and not as its only or most representative instance. A fuller study would need to test this argument against other indigenous historians of the same period, a limitation acknowledged here rather than resolved. Finally, it is worth taking seriously the possibility that Peter and Peter’s framing of the Nairs as “oppressors” owes as much to a contemporary liberationist political commitment as to any direct inheritance from colonial ethnography. These two explanations are not, in fact, mutually exclusive: this study’s argument is precisely that colonial categories of “oppressor” and “oppressed” have become available as a political vocabulary for indigenous liberationist projects that are otherwise entirely non-colonial in their aims, which is one of the more troubling implications of describing this process as epistemicide rather than simple continuity. Peter and Peter’s liberationist commitment to the Nadar cause does not stand outside the colonial genealogy this paper traces; it is one of the routes by which that genealogy continues to operate in the present.

## Conclusion

The study offers a comprehensive investigation into how the identity of the Nair community was manufactured and sustained across colonial, neo-colonial, and native historical registers. This case functions as a paradigmatic instance of the epistemic dynamics through which colonial frameworks are reproduced across the wider landscape of South Asian historiography, catalyzed by specific historiographical shifts through a contrapuntal reader of the three primary texts, the research exposes the persistent resonance of colonial frameworks. Utilizing Edward Said’s methodology of “contrapuntal reading” the study reveals how these texts reinforce imperial hierarchies and suppress indigenous epistemologies. It is important to acknowledge the methodological limits of the study at the level of contrapuntalism. A fully contrapuntal reading would require the recovery of vernacular counter voices, which is the Malayalam language reform periodicals, the *taravad* legal case records, the oral histories of matrilineal households, that colonial scholarship displaced and that remain largely unincorporated into academic historiography. The present study operates at the level of the textual archive, demonstrating how the colonial framework was reproduced across three historiographical moments. The recovery of the suppressed counter-discourse, what Menon identifies as the “local ways of remembering the past” that British historiographical authority marginalized, remains the necessary next stage of the decolonial project this initiates (Menon, 2015, pp. 64-65). In this sense, the study is best understood as a genealogical rather than enacting its reversal. The enactment of a genuinely contrapuntal historiography of the Nairs would require engagement with archival and oral sources of the kind that Arunima’s work exemplifies. It is a project that this study identifies as both necessary and urgent.

The reliance on a Western lens across all three texts highlights the enduring power of the colonial archive over the indigenous past. A contrapuntal analysis of Fawcett’s *Nayars of Malabar* reveals the specific moment when these stereotypes were codified into a formal anthropological “truth”, framing matriliny as “primitive” and *sambandham* as “concubinage”. Here, Fawcett created a framework that stripped the Nair community of its social legitimacy. The transition from Fawcett’s ethnography to Jeffrey’s archival study marks the academic institutionalization of colonial tropes. Peter’s subsequent revisionism functions as a site of “entangled history”, where these stereotypes are recirculated under the guise of indigenous scholarship. This analysis, supplemented by Hayden White’s theory of “emplotment”, exposes the rhetorical and narrative architecture of these historical writings. History, in this view, is not a transparent window into the past but a linguistic construct. White’s framework confirms that history is fundamentally a narrative construction; through emplotment, colonial histories are structured to follow identifiable plot lines, proving they are concisely shaped narratives rather than neutral, passively recorded facts.

The theoretical convergence of Whites’s “emplotment”, Said’s “contrapuntal reading”, and Santos “epistemicide” provides a productive analytical framework for tracing the structural mechanisms through which epistemologies were delegitimized, institutionalized in academic form and finally internalized by indigenous historians. Cohn’s analysis of colonial investigative modalities established the institutional conditions under which these processes occurred. Chakrabarty’s critique of historicism explains the temporal logic, which is the “waiting room of history” through which the colonial narrative of Nair “decline” was naturalized as a developmental inevitability rather than a political construction. Together, these frameworks demonstrate that epistemicide was not a single event of colonial imposition but a recursive process, repeated and reinforced across distinct historiographical moments from Fawcett’s ethnographic survey to Jeffrey’s academic monograph to Peter’s indigenous revisionism. This analysis contributes to the broader project of decolonizing South Asian historiography not by recovering an unmediated indigenous past, but by making visible the mechanisms through which colonial authority was reproduced as historical truth.

A contrapuntal reading is proposed as the essential lens for drafting and interpreting Indian communal history, as it explicitly exposes the dominant, clandestinely influential voices embedded within the historiography. Systematic analysis reveals that the Western gaze is deeply ingrained in Indian historical narratives, having subtly infiltrated and become a foundational element of contemporary community identity. The application of White’s emplotment, Said’s contrapuntalism, and Santos’s epistemicide serves to destabilize the colonial archive, revealing the systematic delegitimization of the Nair social logic. This theoretical synthesis facilitates the reclamation of indigenous epistemologies, positioning them as primary rather than derivative intellectual frameworks. In doing so, the study intervenes in colonial and postcolonial discourse, illustrating how historical narratives function as instruments of ongoing epistemic restoration and advocating for a shift toward more critically reflexing historiographical practices.

Santos (2014, 2018) develops the concept of epistemicide with reference to colonial and postcolonial knowledge systems across the Global South generally, rather than to the Nair case specifically; applying his framework here, the epistemicide documented in the context of the Nair community should be understood as one local instance of what Santos identifies as a pervasive condition across the Global South, rather than as a phenomenon Santos himself describes. It is a structural feature of colonial epistemic inheritance that postcolonial scholarship has yet fully dismantled. Future research in this vein must move beyond the textual archive to engage the vernacular counter-discourse that colonial historiography displaced like Malayalam language reform periodicals, the *taravad* legal records, and the oral testimonies of matrilineal households that constitute the suppressed other side of the historical record examined here. Only by recovering and centering these marginalized epistemologies (rather than simply critiquing their erasure) can the decolonial project that this study initiates be brought to completion. In this sense, the present analysis does not close the question of Nair historiography but opens it. It establishes the genealogy of epistemic distortion that any future contrapuntal account of the community must work against.
